# Supplementary figures and images for: Elevated TGF-β1 impairs synaptic and cognitive function through activation of Smad2/3-Sp1 pathway in AngII-related hypertension
Source: EMBO Rep. 2025 May 27;26(12):3162–86. doi: 10.1038/s44319-025-00470-0 (PMC12187917; doi:10.1038/s44319-025-00470-0)

2C

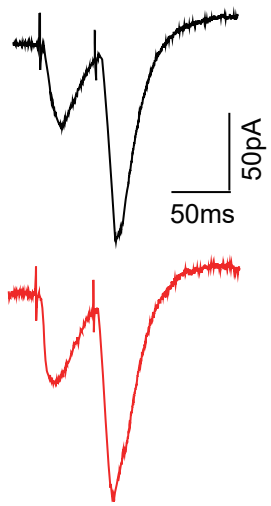

2F

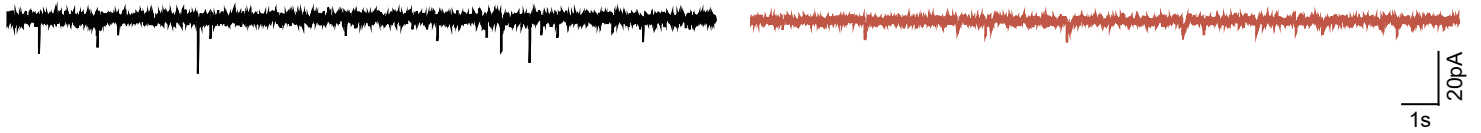

2H

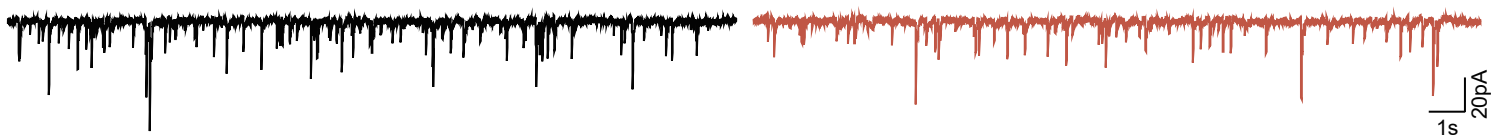

Supplement: Supplementary file 3 — Source data Fig. 2 [file 44319_2025_470_MOESM3_ESM.zip › Figure 2 Source Data/2C-2F-2H/2C-2F-2H.pdf]

CTR

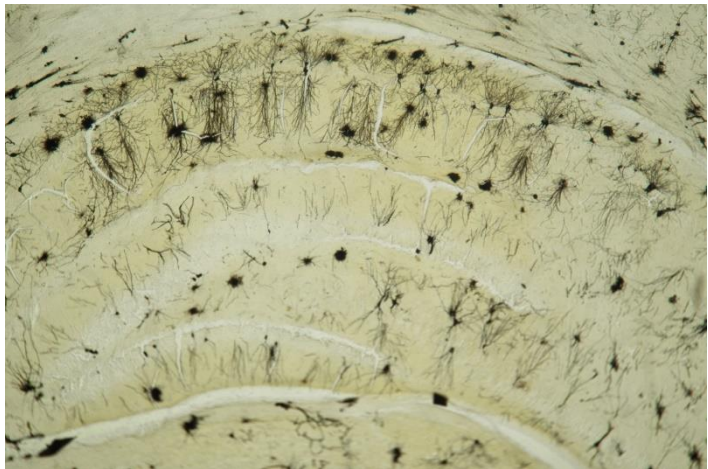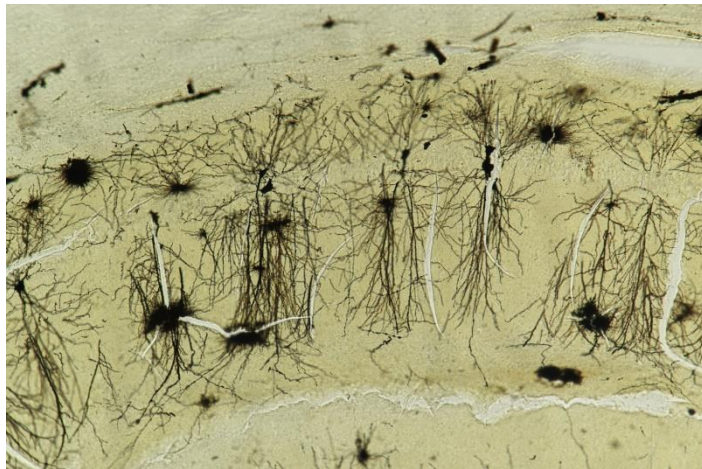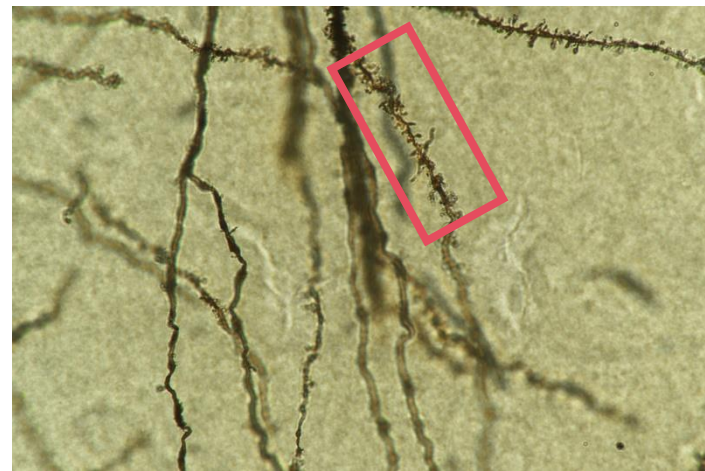

Ang II

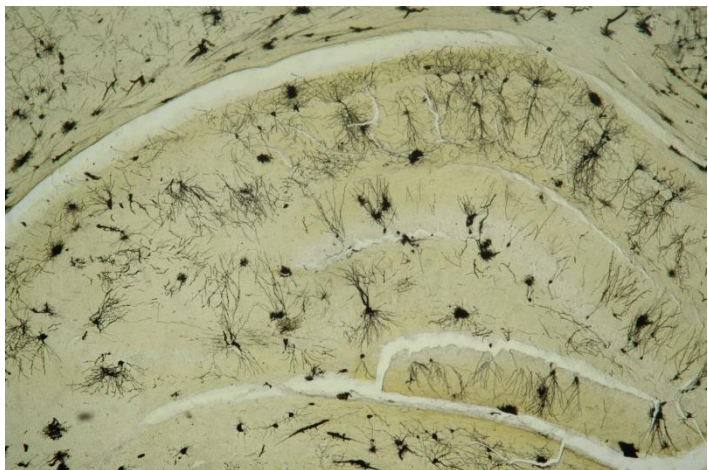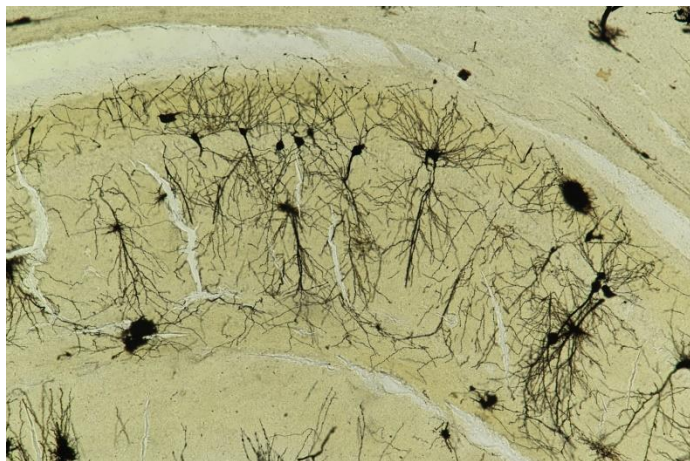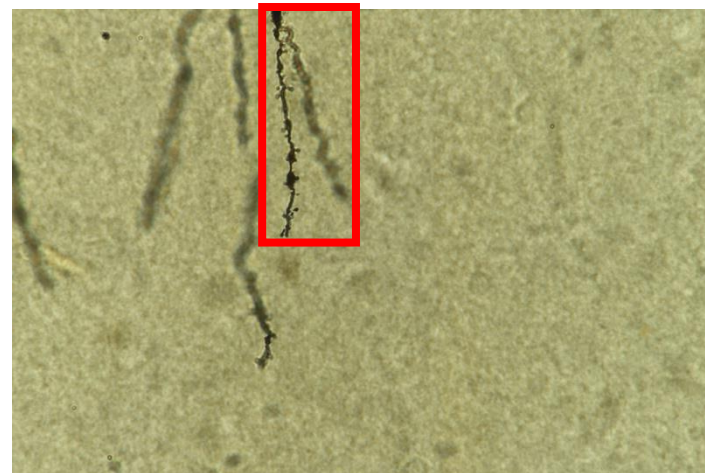

Supplement: Supplementary file 3 — Source data Fig. 2 [file 44319_2025_470_MOESM3_ESM.zip › Figure 2 Source Data/2J/2J.pdf]

3E

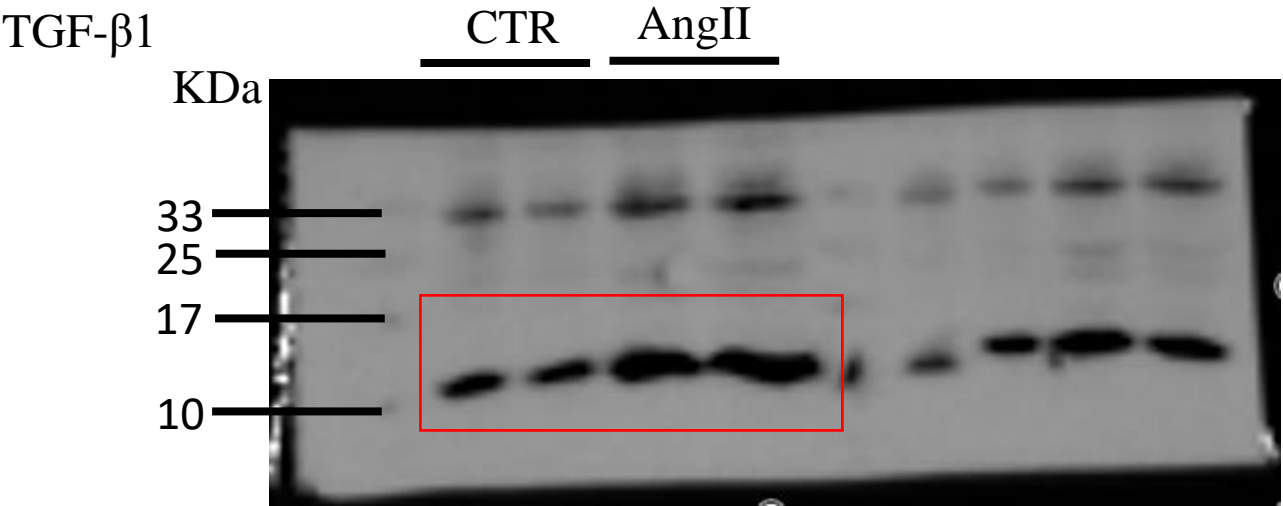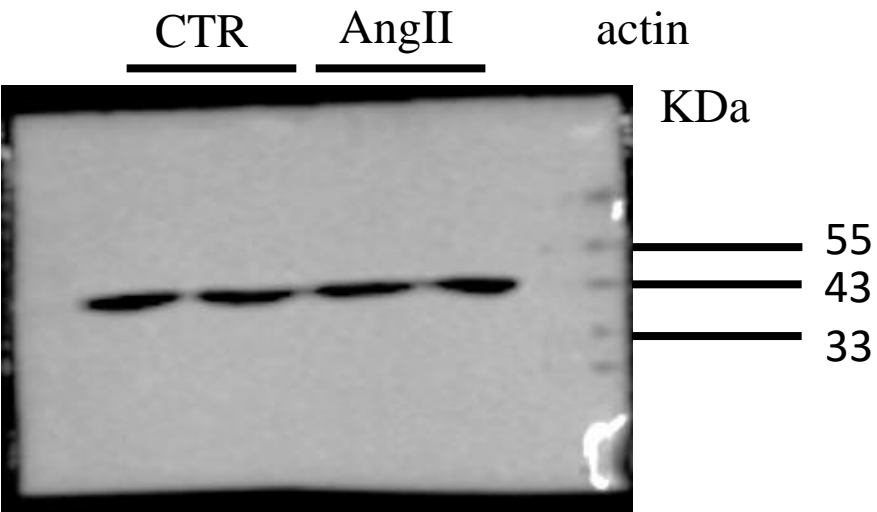

Supplement: Supplementary file 4 — Source data Fig. 3 [file 44319_2025_470_MOESM4_ESM.zip › Figure 3 Source Data/3E/3E.pdf]

3F

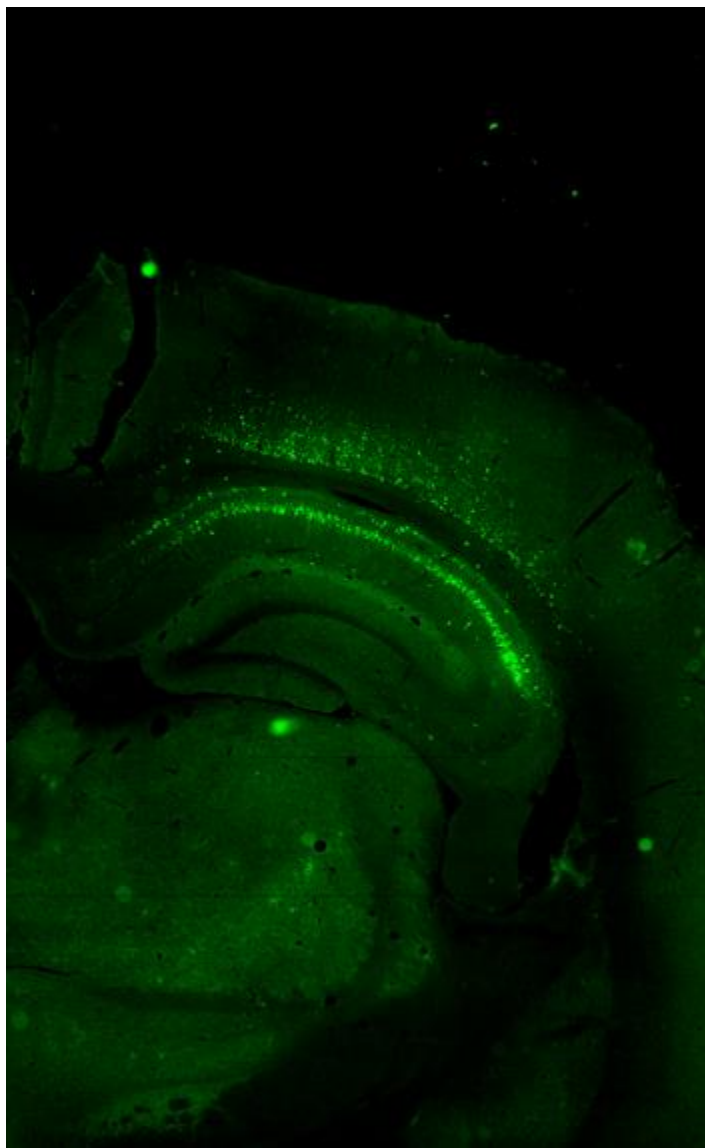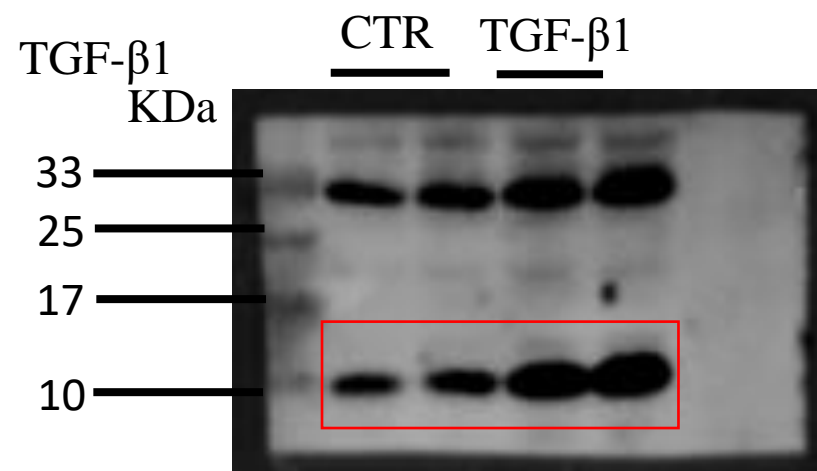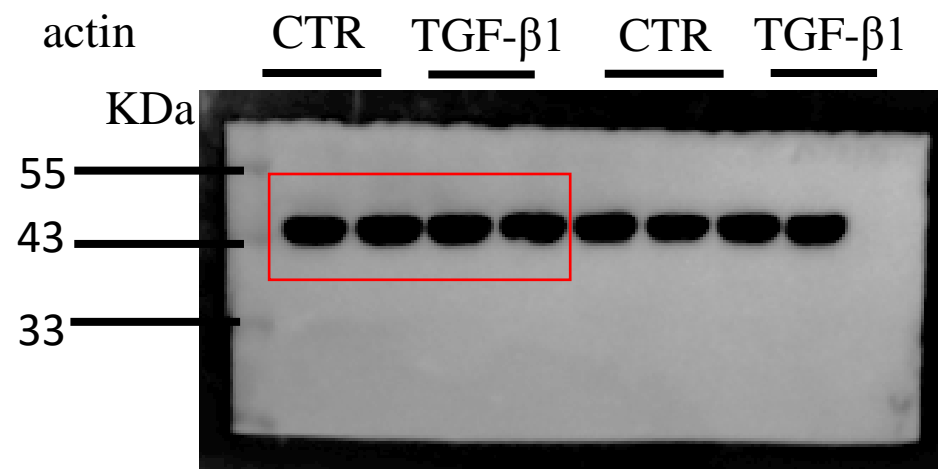

Supplement: Supplementary file 4 — Source data Fig. 3 [file 44319_2025_470_MOESM4_ESM.zip › Figure 3 Source Data/3F/3F.pdf]

4J

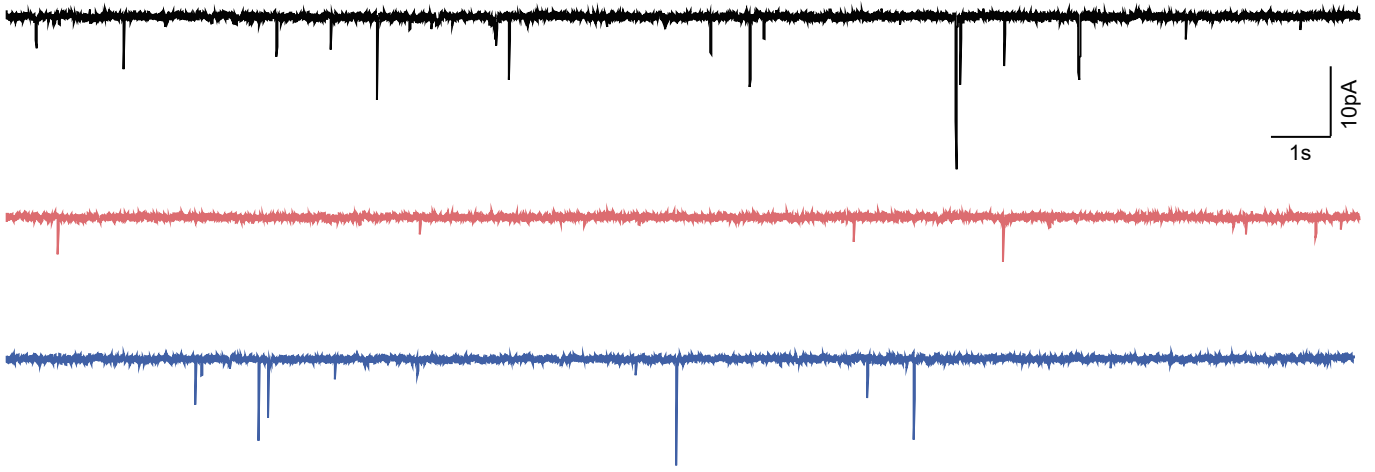

Supplement: Supplementary file 5 — Source data Fig. 4 [file 44319_2025_470_MOESM5_ESM.zip › Figure 4 Source Data/4J/4J.pdf]

CTR

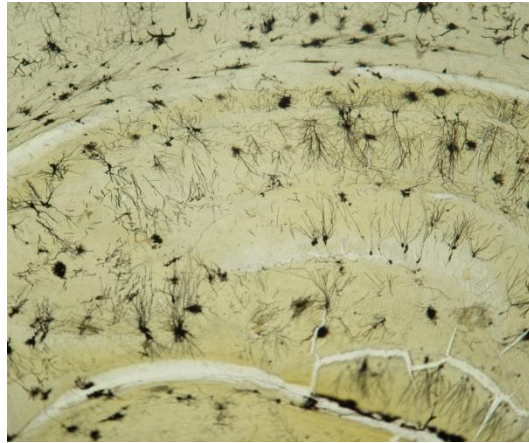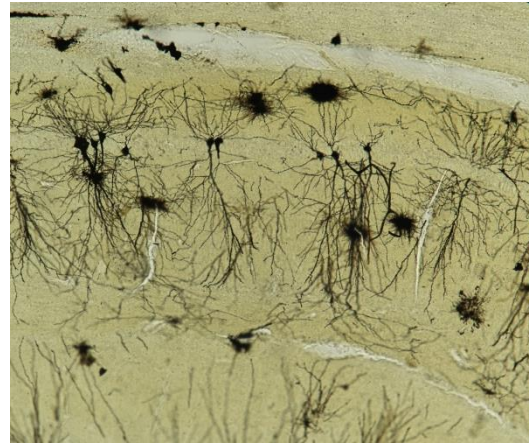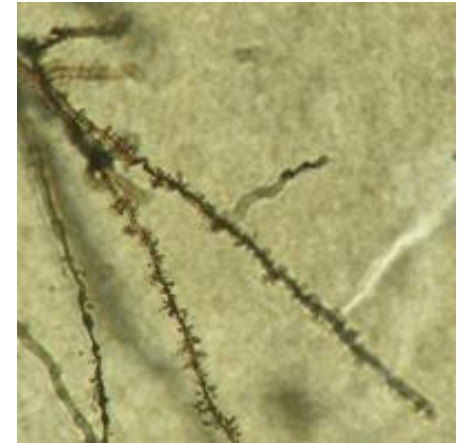

Ang II

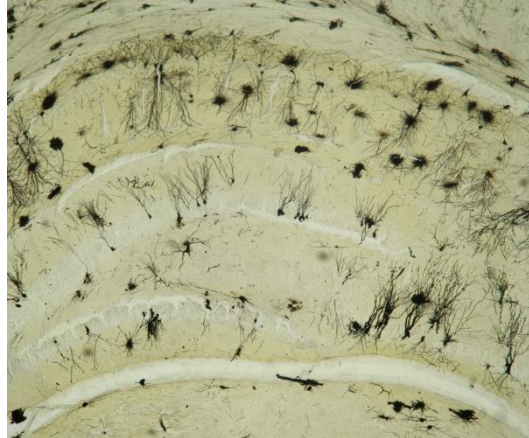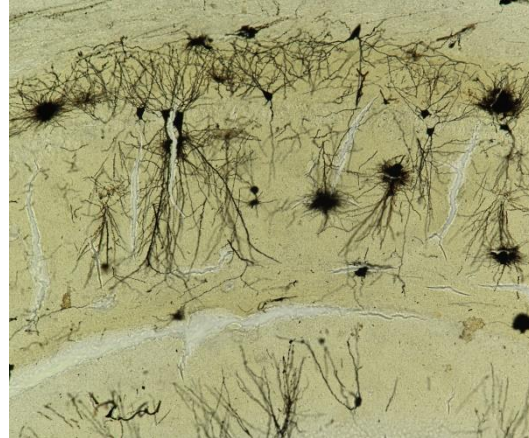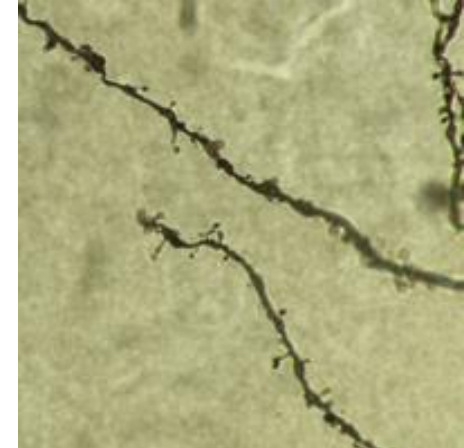

Ang II +Sh-TGF  $\beta$ 1

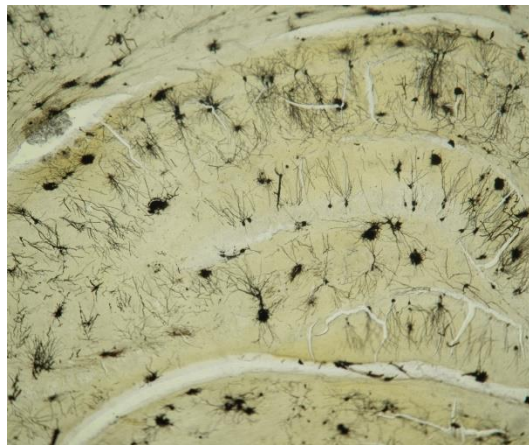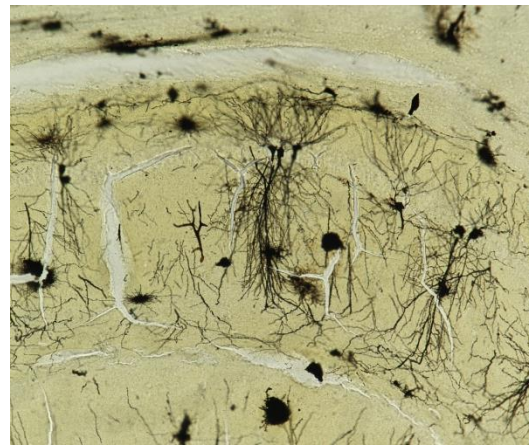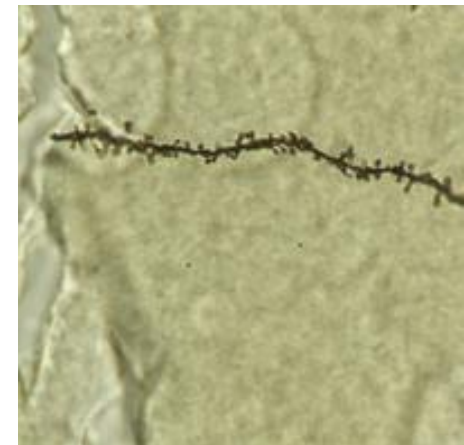

Supplement: Supplementary file 5 — Source data Fig. 4 [file 44319_2025_470_MOESM5_ESM.zip › Figure 4 Source Data/4M/4M.pdf]

**Figure 5A**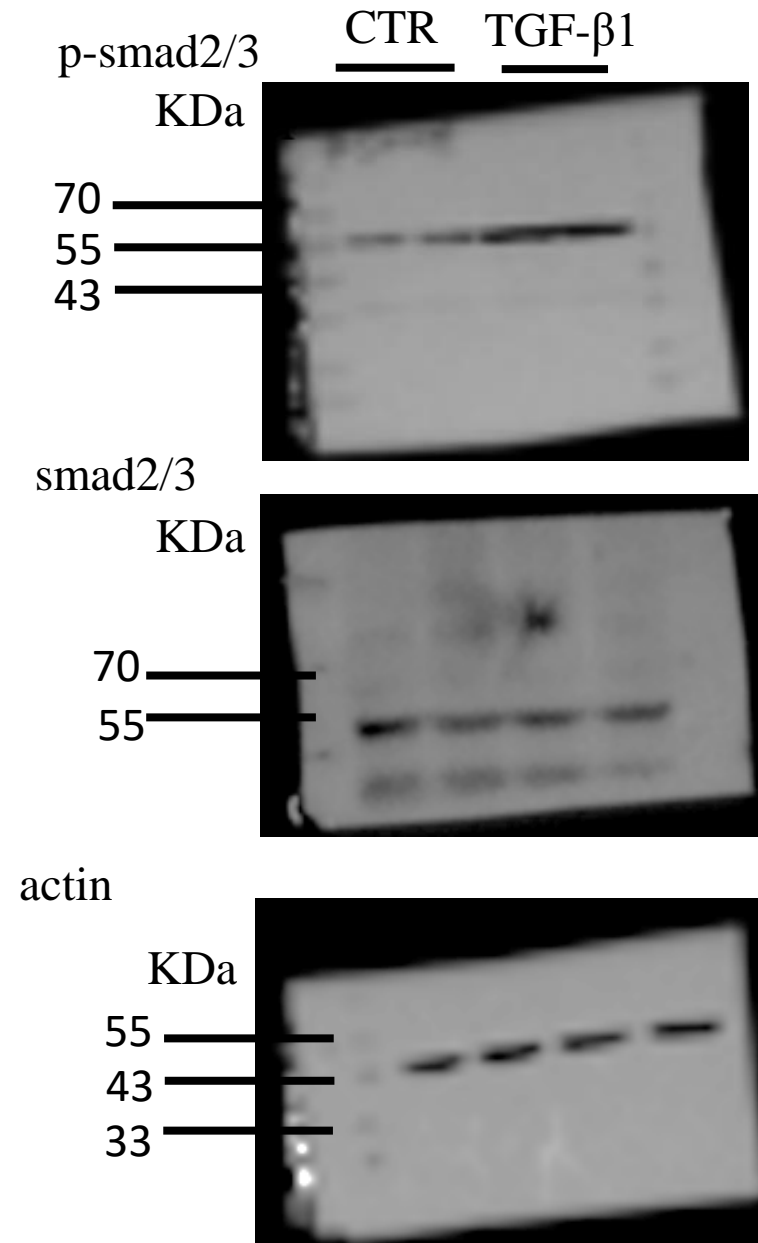**Figure 5C**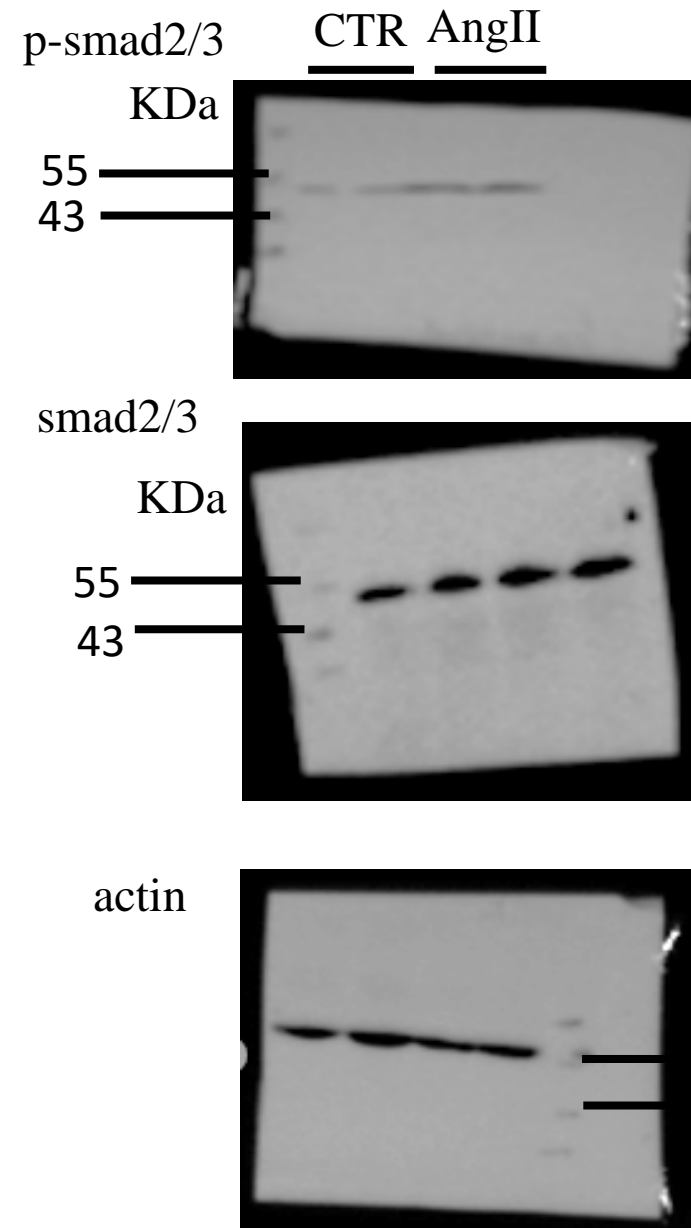**Figure 5E**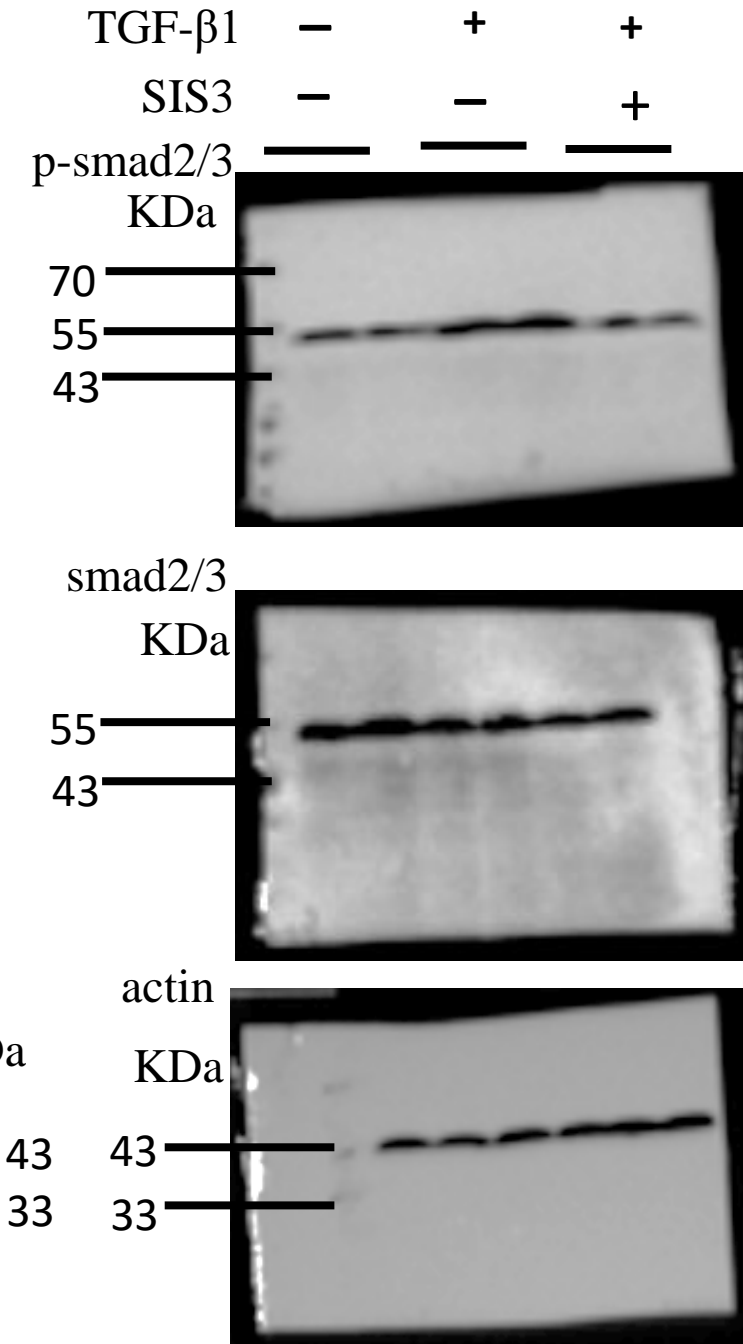

Figure 5J

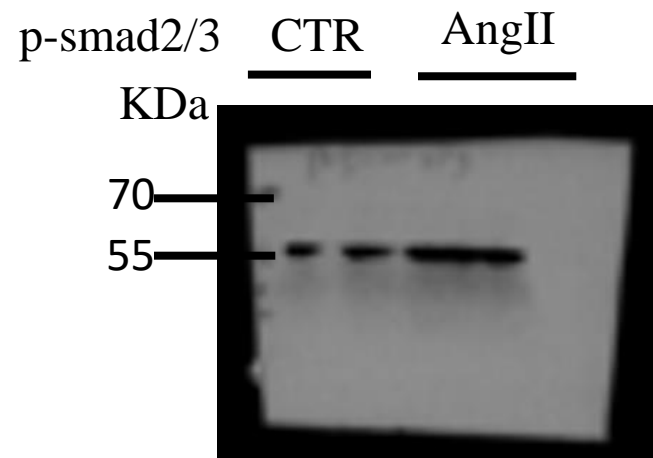

Figure 5L

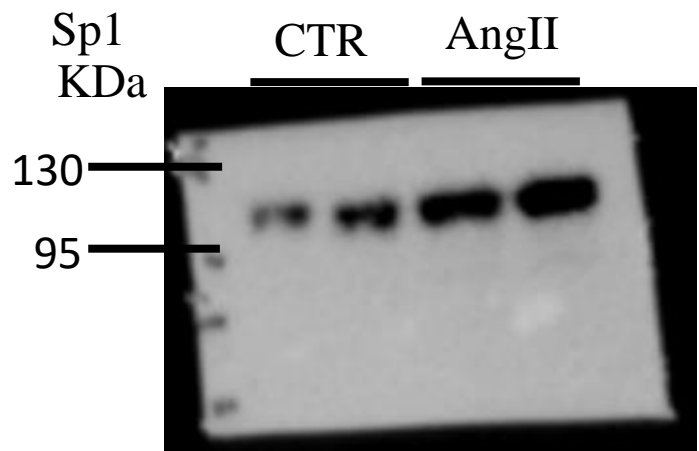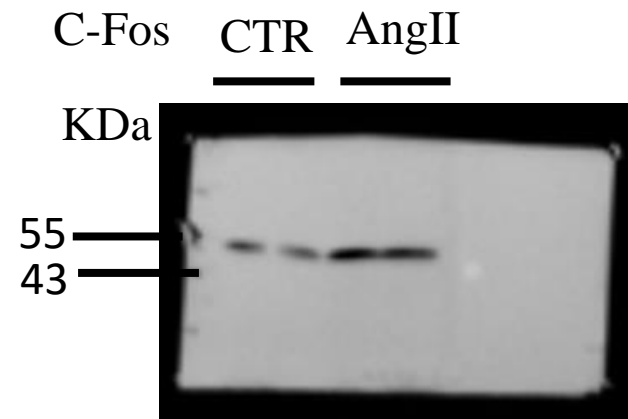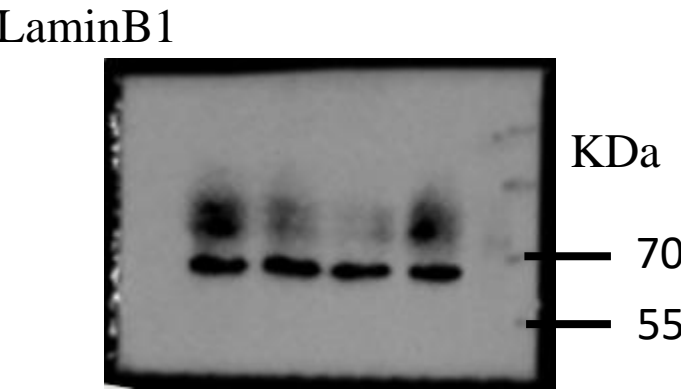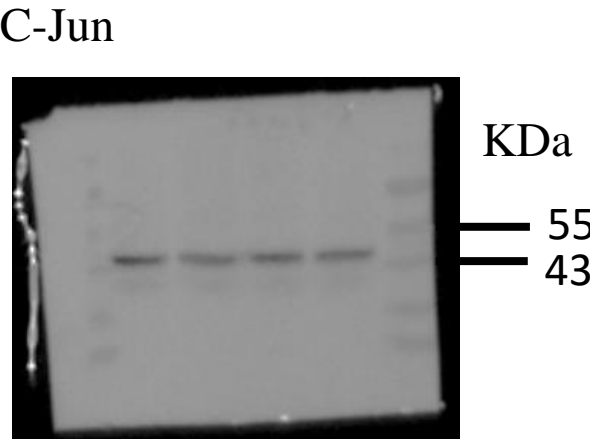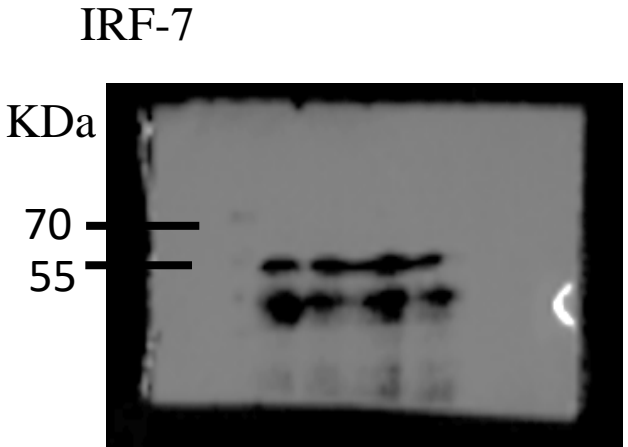

Supplement: Supplementary file 6 — Source data Fig. 5 [file 44319_2025_470_MOESM6_ESM.zip › Figure 5 Source Data/5A-5C-5E-5J-5L/5A-C-E-J-L.pdf]

5G

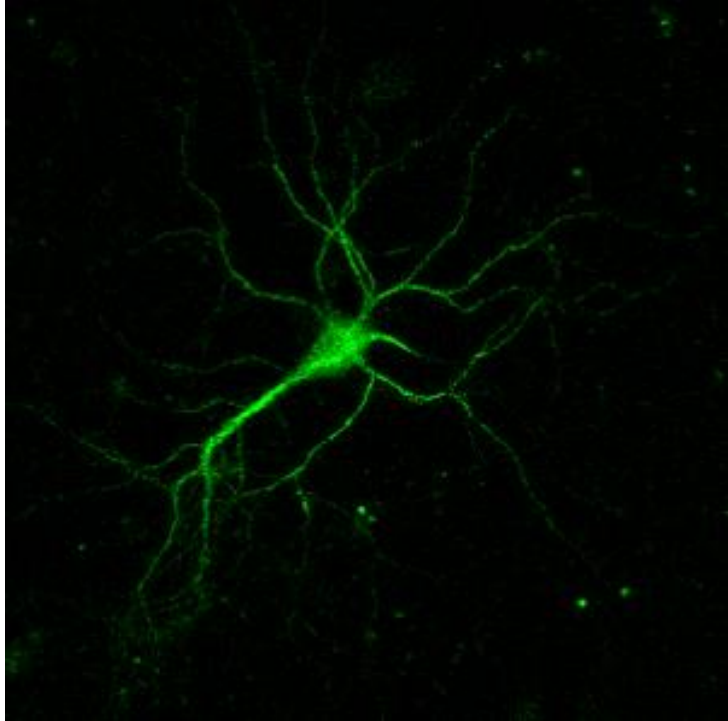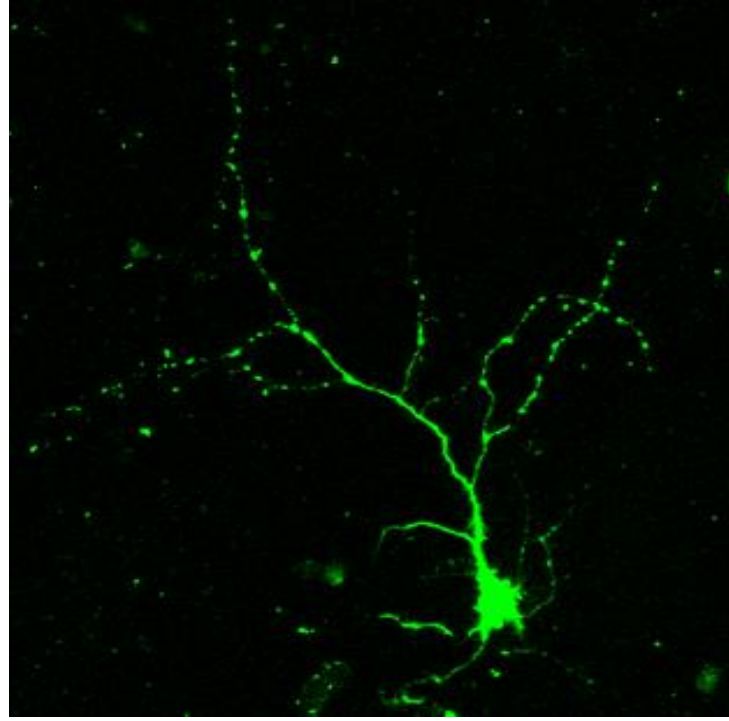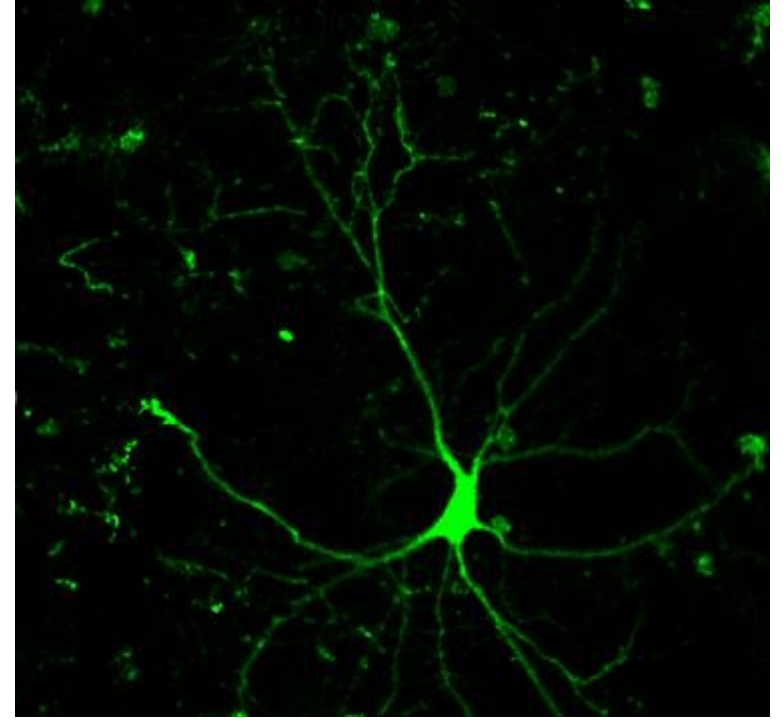

Supplement: Supplementary file 6 — Source data Fig. 5 [file 44319_2025_470_MOESM6_ESM.zip › Figure 5 Source Data/5G/5G.pdf]

6D

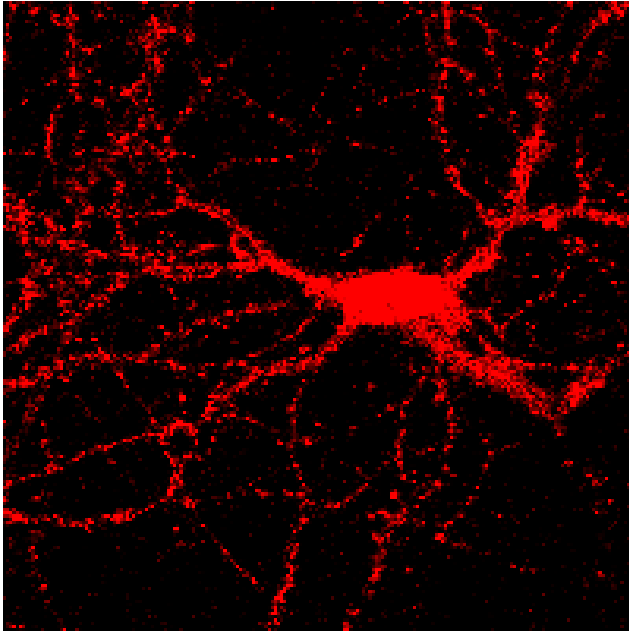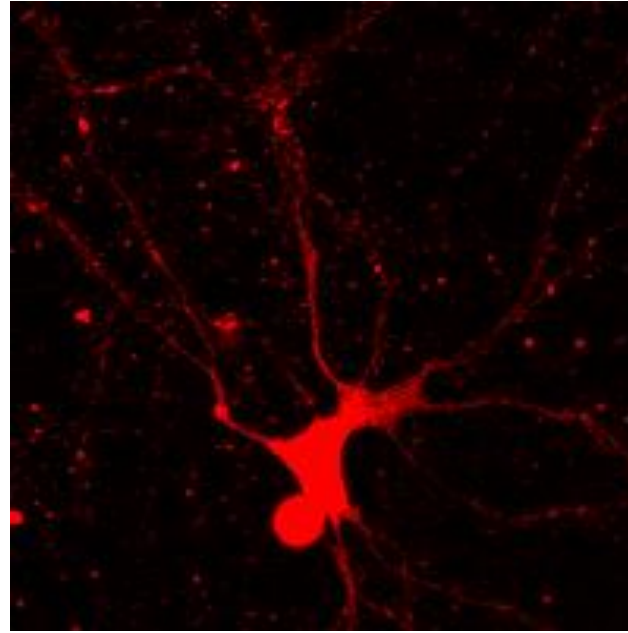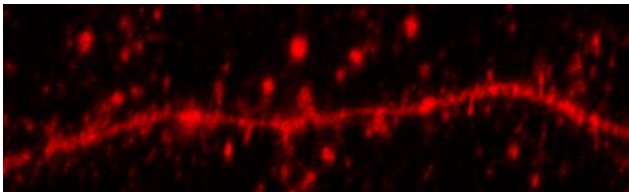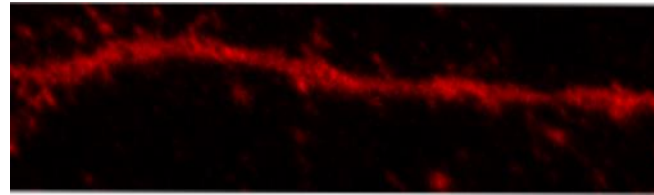

Supplement: Supplementary file 7 — Source data Fig. 6 [file 44319_2025_470_MOESM7_ESM.zip › Figure 6 Source Data/6D/6D.pdf]

7A

Vector

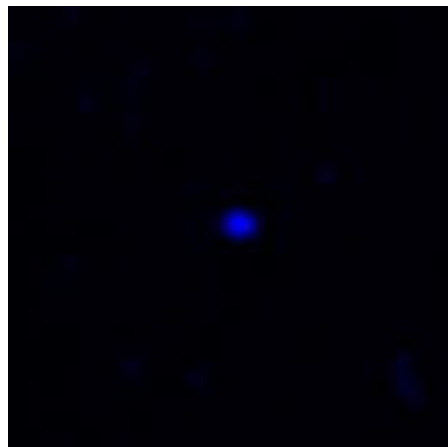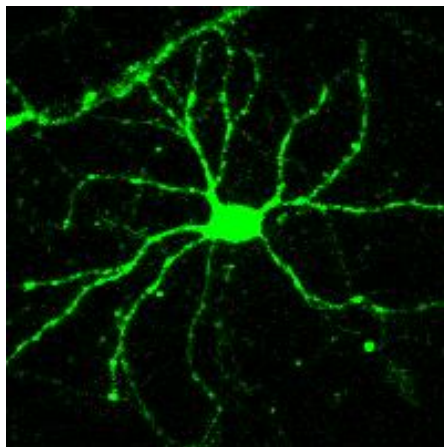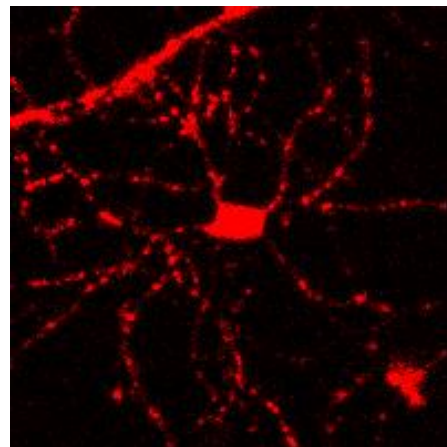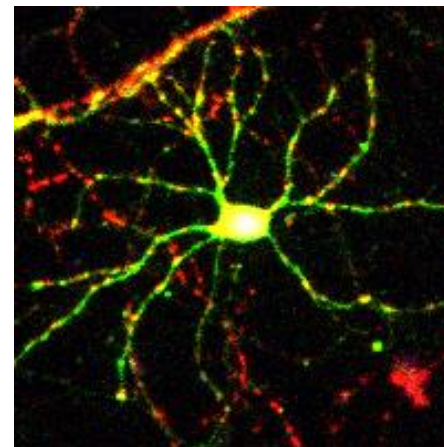

TGF- $\beta$ 1+sh-vector

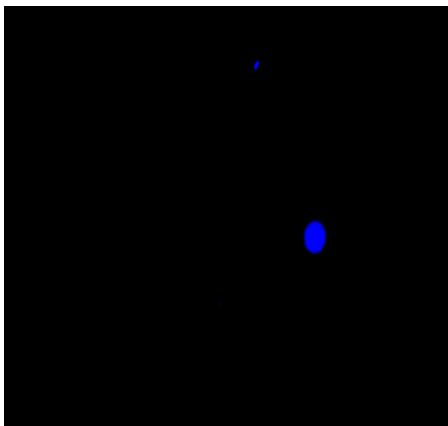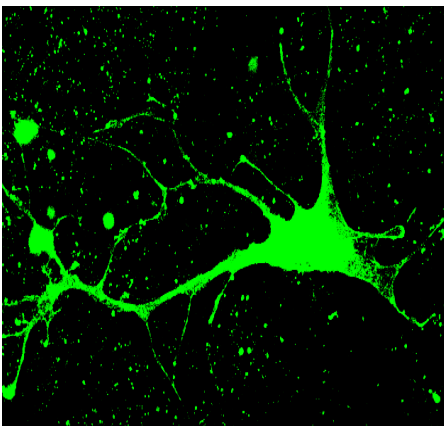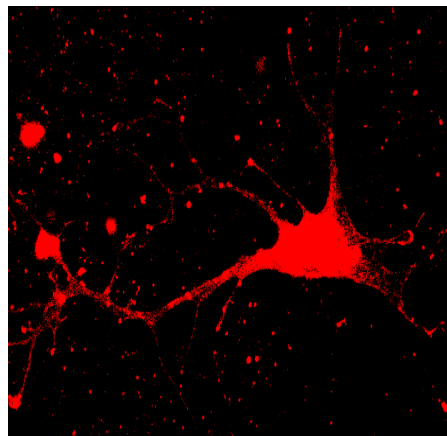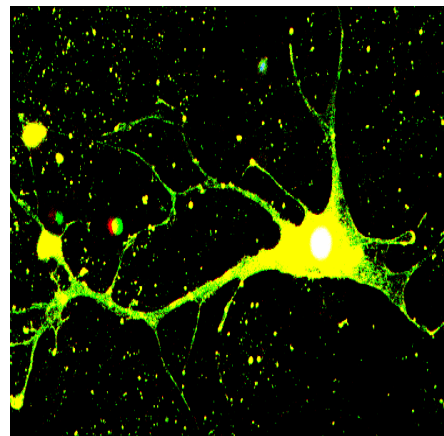

TGF- $\beta$ 1+sh-Sp1

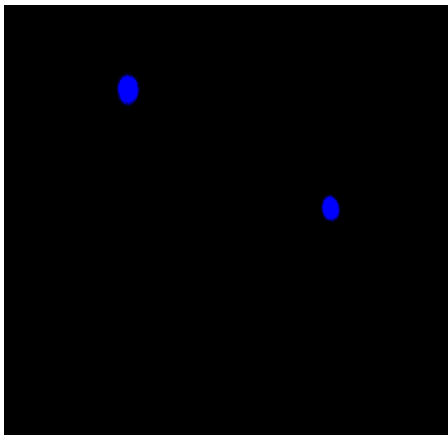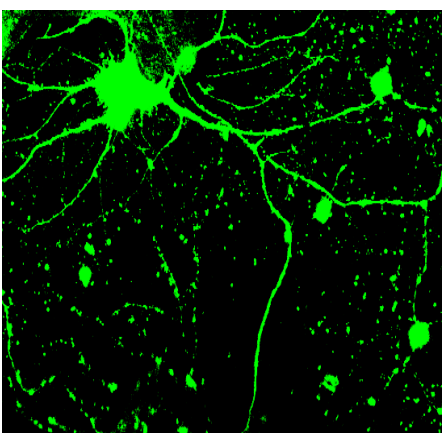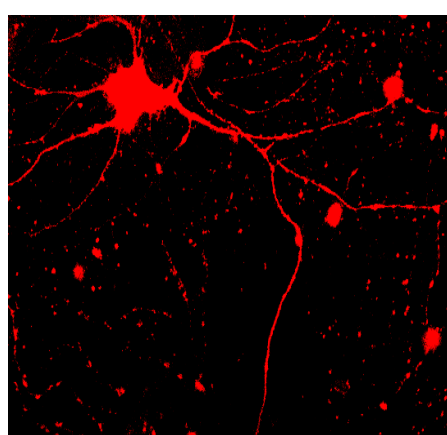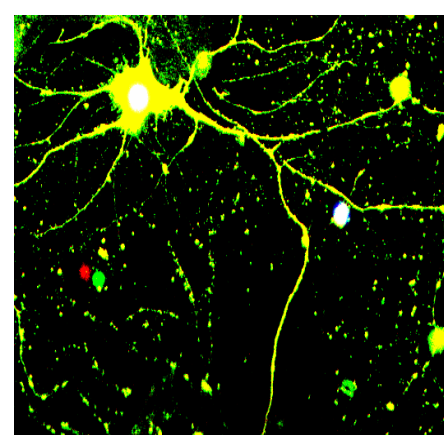

Supplement: Supplementary file 8 — Source data Fig. 7 [file 44319_2025_470_MOESM8_ESM.zip › Figure 7 Source Data/7A/7A.pdf]

7D

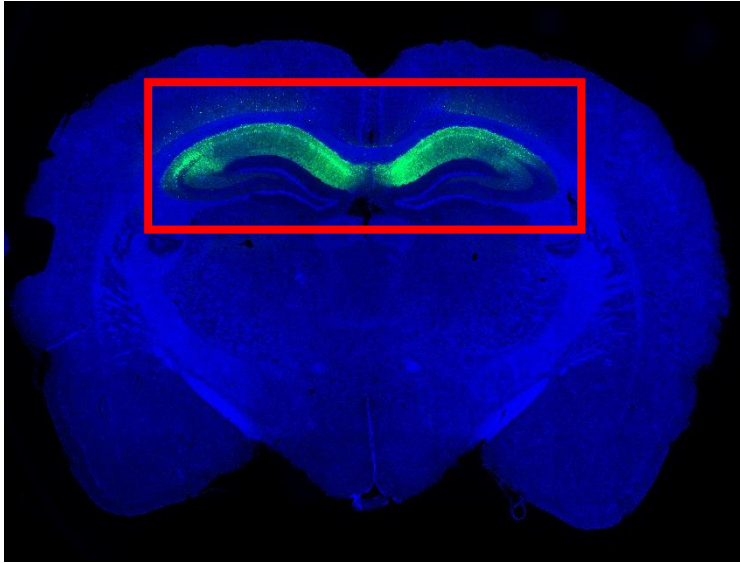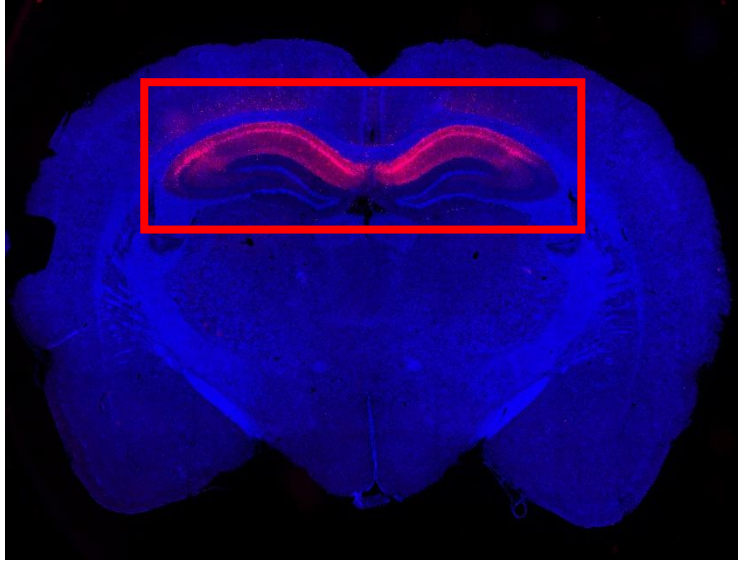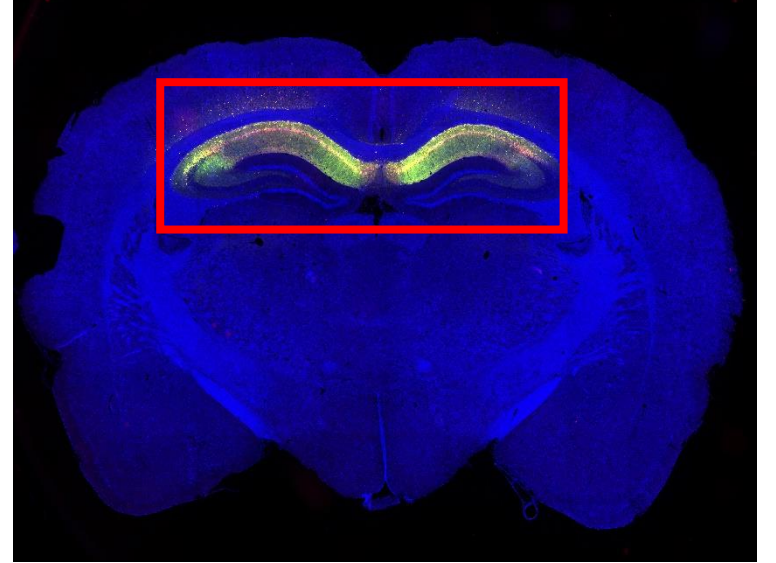

Supplement: Supplementary file 8 — Source data Fig. 7 [file 44319_2025_470_MOESM8_ESM.zip › Figure 7 Source Data/7D/7D.pdf]

7L

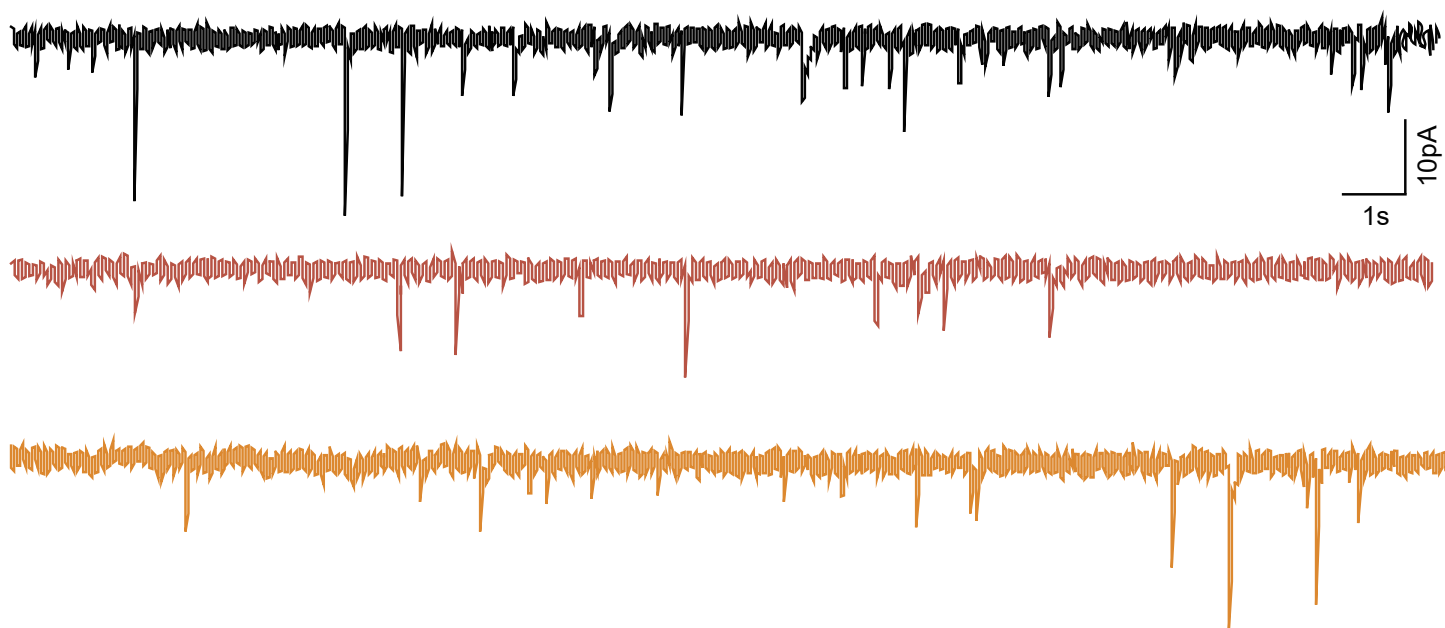

Supplement: Supplementary file 8 — Source data Fig. 7 [file 44319_2025_470_MOESM8_ESM.zip › Figure 7 Source Data/7L/7L.pdf]

1A

Hippocampus cross section

---

Brain-coronal

dorsal

ventral

CTR

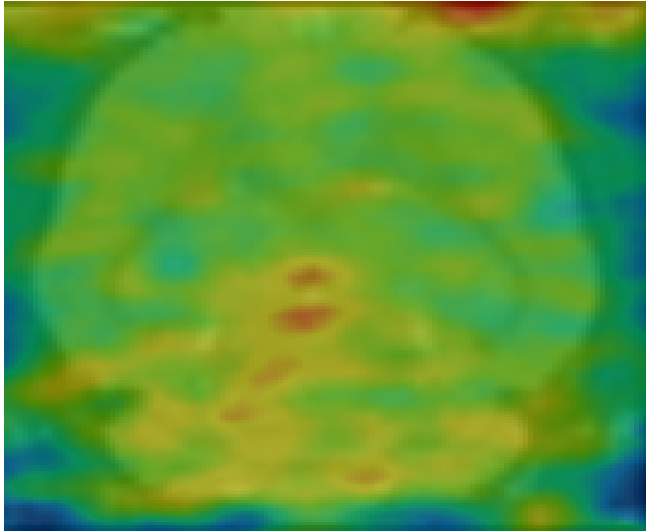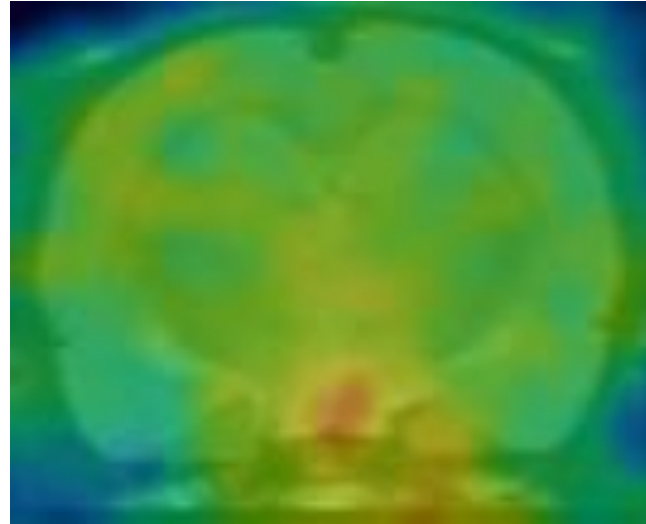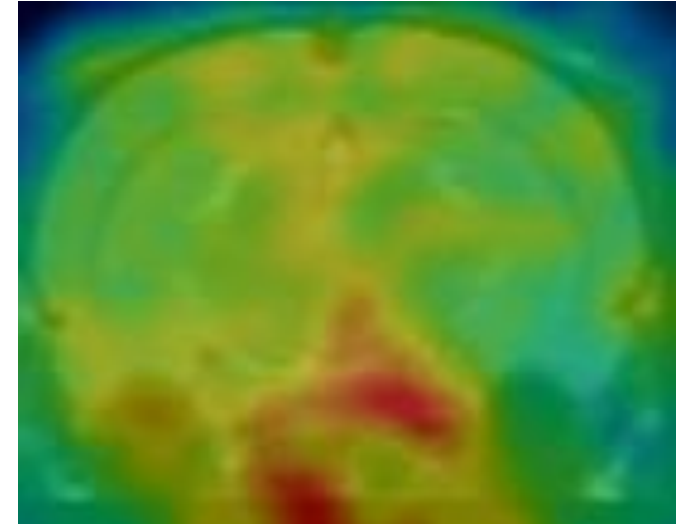

AngII

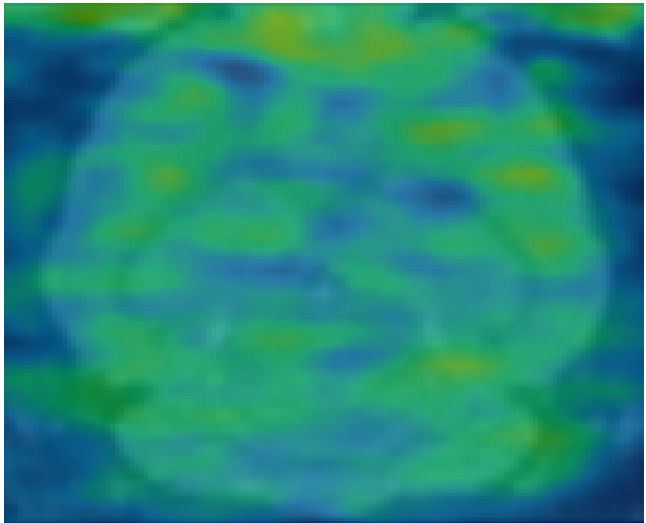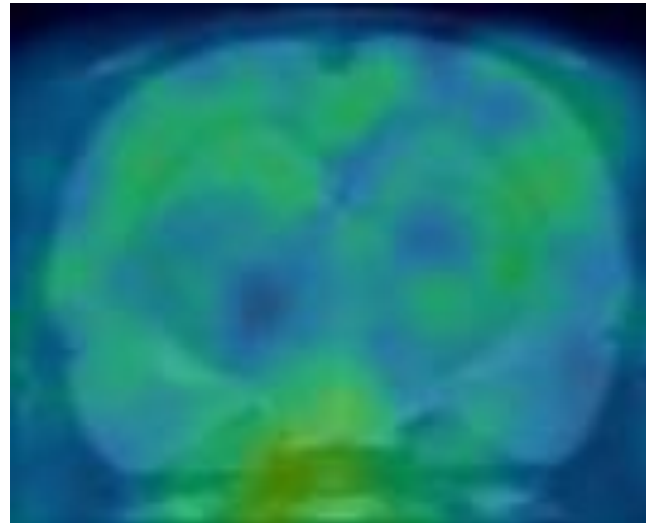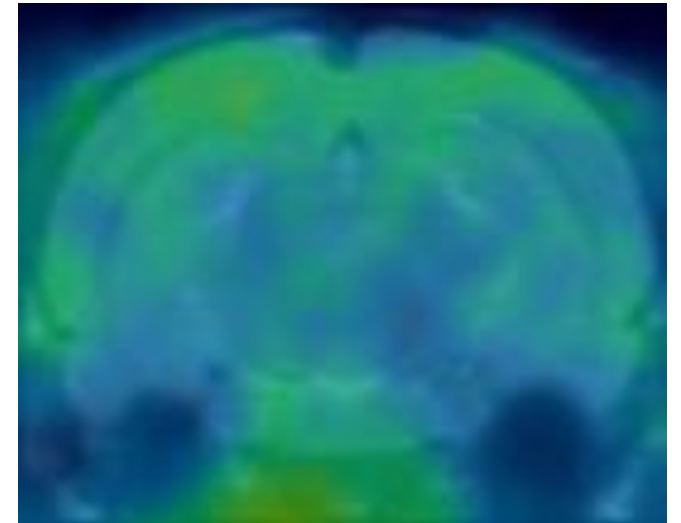

Supplement: Supplementary file 9 — Figure EV1 Source Data [file 44319_2025_470_MOESM9_ESM.zip › Figure Expanded view 1 Source Data/1A/EV1A.pdf]

# Figure EV3D

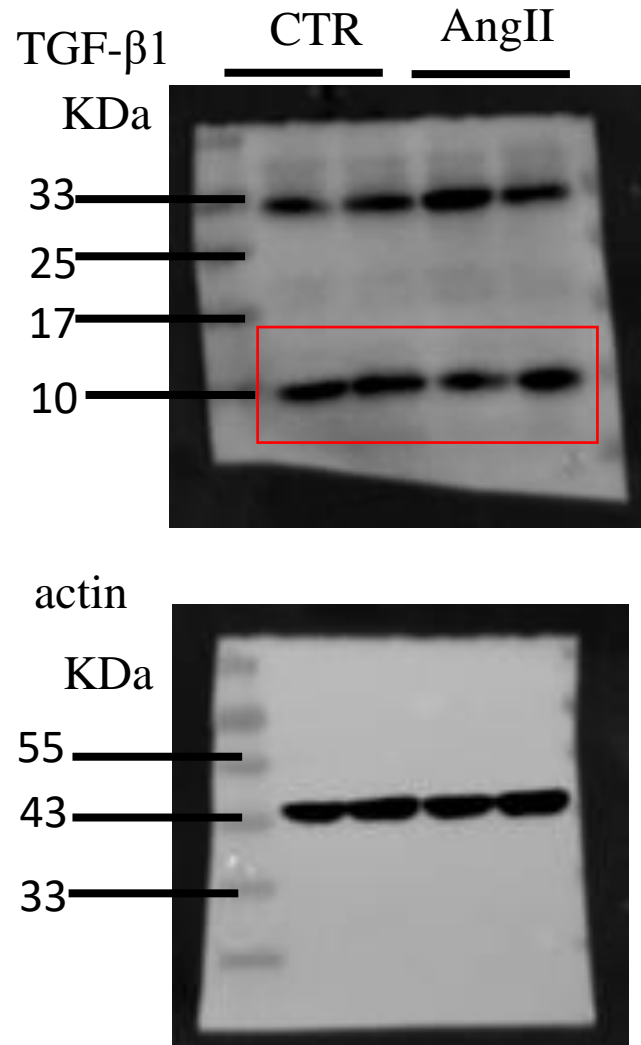

# Figure EV3F

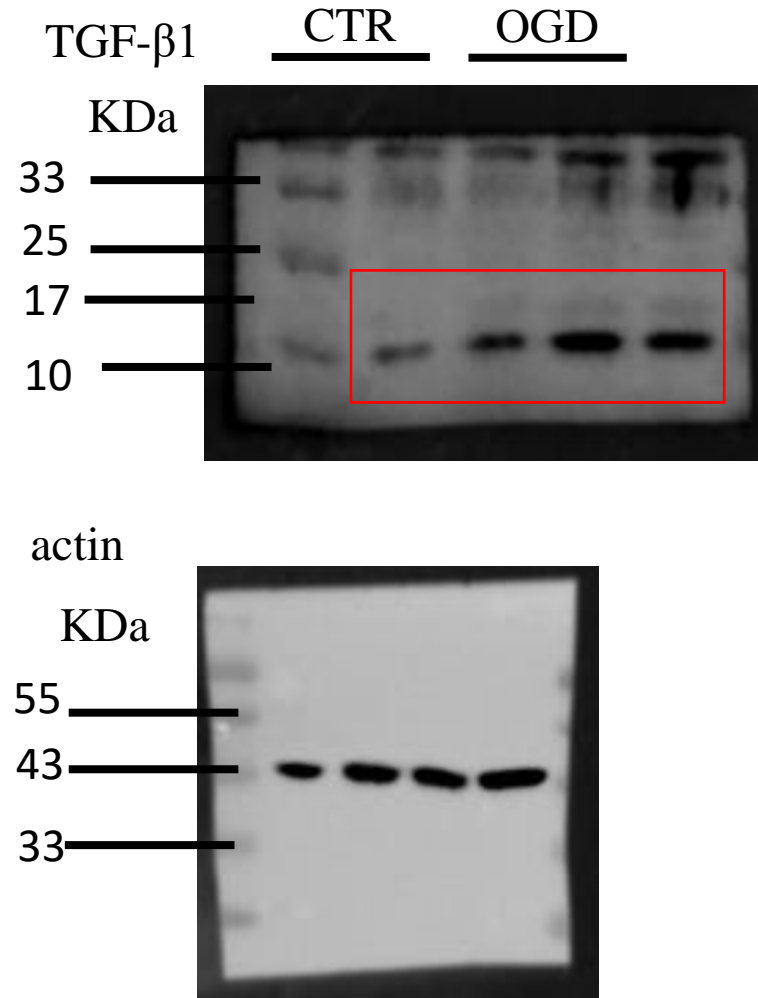

# Figure EV3H

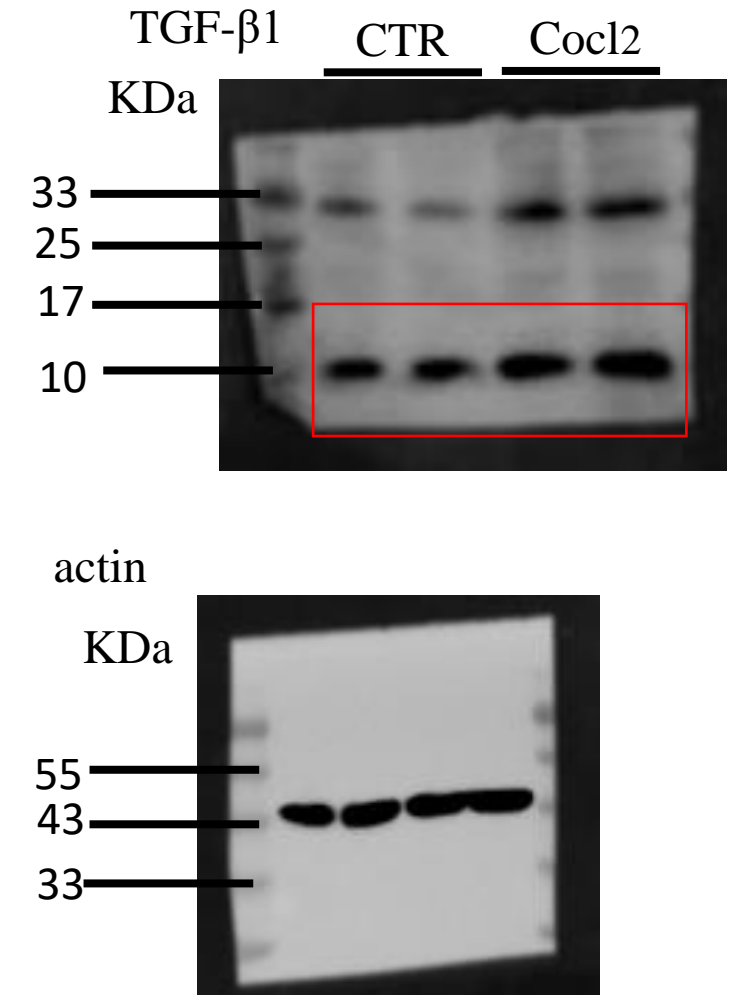

Supplement: Supplementary file 10 — Figure EV 3 Source Data [file 44319_2025_470_MOESM10_ESM.zip › Figure Expanded view 3 Source Data/3D-3F-3H/3D-F-H.pdf]

4A

Brain coronal

Hippocampus cross section

CTR

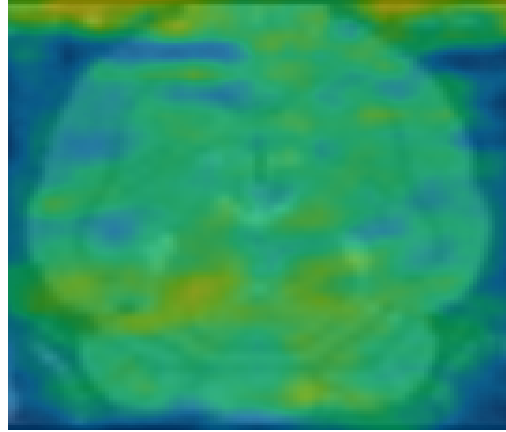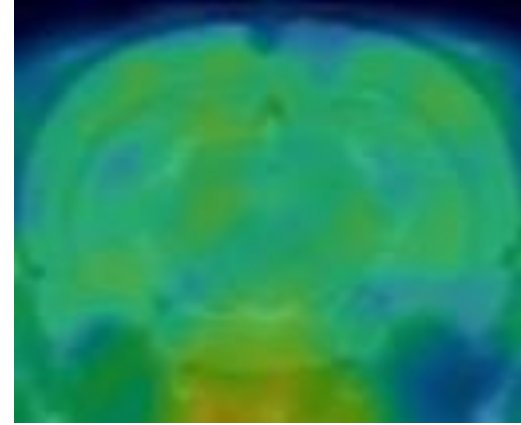

AngII

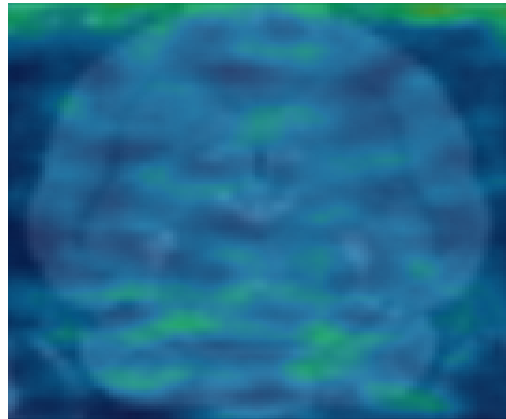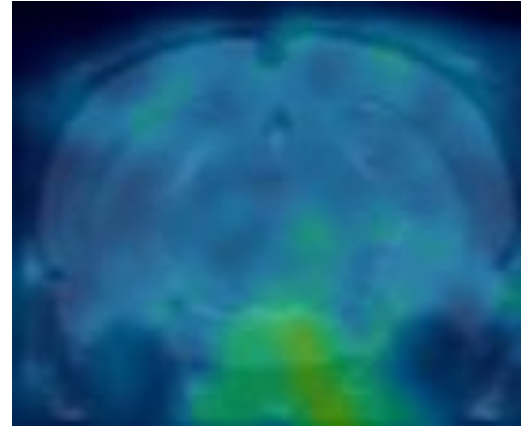

AngII+Sh-TGF- $\beta$ 1

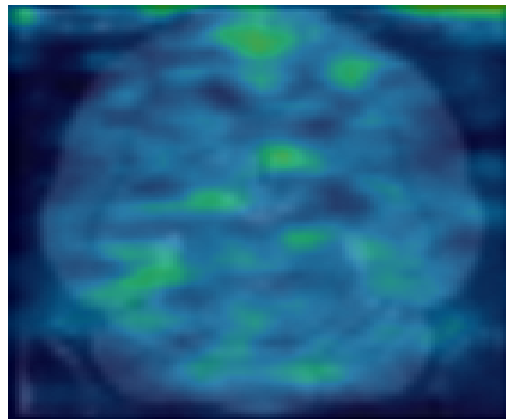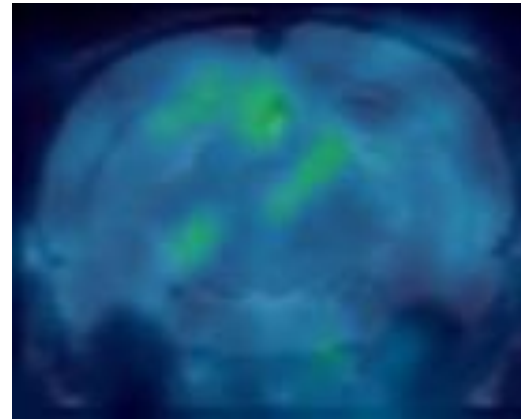

Supplement: Supplementary file 11 — Figure EV4 Source Data [file 44319_2025_470_MOESM11_ESM.zip › Figure Expanded view 4 Source Data/4A/EV4A.pdf]
