# Supplementary material for: Elevated TGF-β1 impairs synaptic and cognitive function through activation of Smad2/3-Sp1 pathway in AngII-related hypertension
Source: EMBO Rep. 2025 May 27;26(12):3162–86. doi: 10.1038/s44319-025-00470-0 (PMC12187917; doi:10.1038/s44319-025-00470-0)
Supplement: Supplementary file 12 — Expanded View Figures [file 44319_2025_470_MOESM12_ESM.pdf]

Expanded View Figures

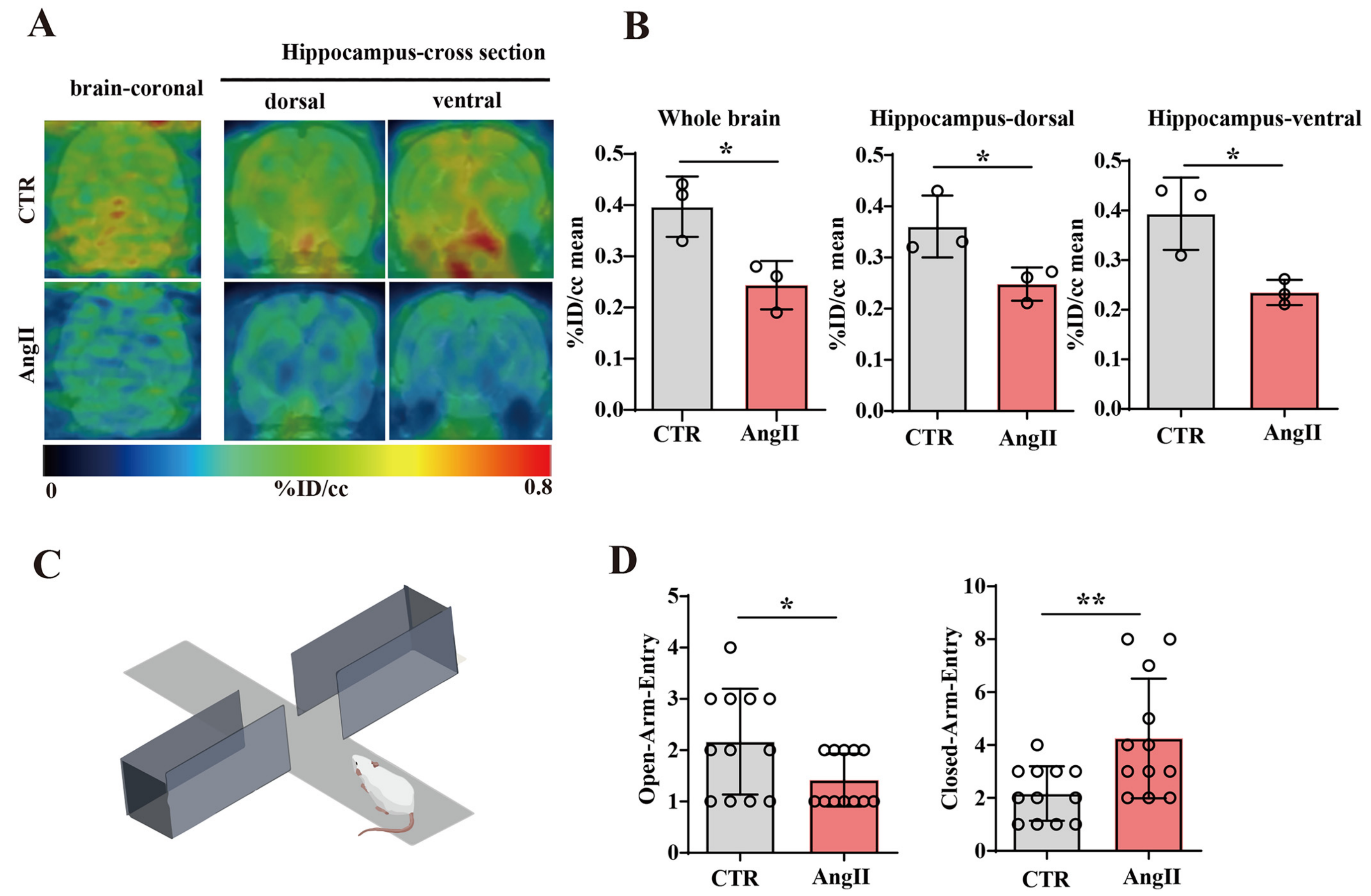

Figure EV1. AngII-related hypertension reduced blood flow and cognitive impairments.

(**A**) The 13N-NH3-PET rat brain dynamic imaging was used to observe the blood flow in the brain and hippocampus. (**B**)The results showed that the whole brain ($P = 0.0246$), the hippocampus dorsal ($P = 0.0472$) and ventral ($P = 0.0236$), ($n = 3$). (**C**, **D**) The high plus maze test was performed to measure the entries to the open ($P = 0.0343$) and closed arms ($P = 0.0082$), ($n = 12$). Data are presented as Mean ± SD. A two-tailed Student's *T* test was used for statistical analysis in (**B**, **D**). $^*P < 0.05$, $^{**}P < 0.01$, versus Control group. Source data are available online for this figure.

 © The Author(s)

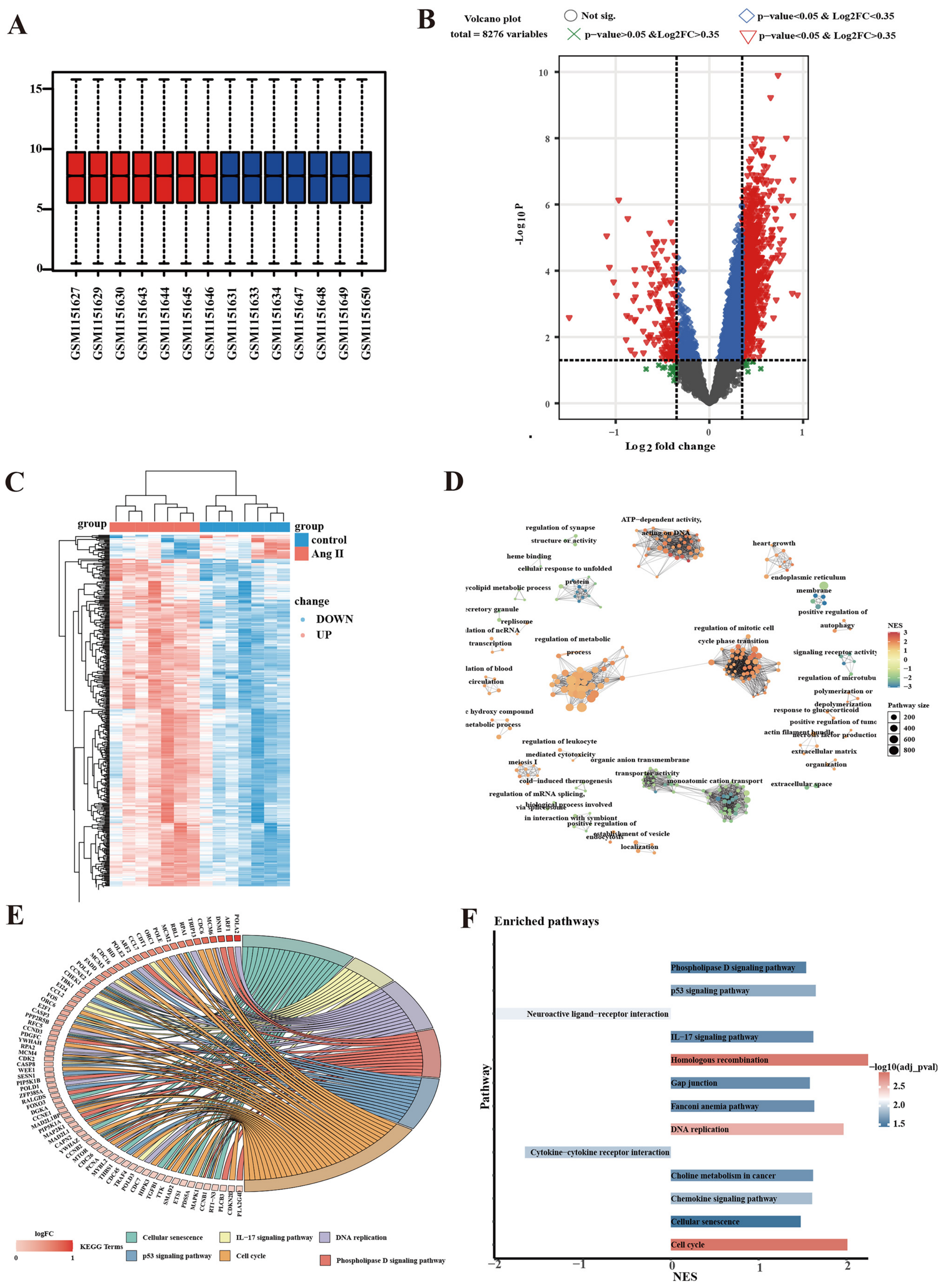
A
GSM1151627
GSM1151629
GSM1151630
GSM1151643
GSM1151644
GSM1151645
GSM1151646
GSM1151631
GSM1151633
GSM1151634
GSM1151647
GSM1151648
GSM1151649
GSM1151650
B
Volcano plot
total = 8276 variables
Not sig.
p−value>0.05 &Log2FC>0.35
p−value<0.05 & Log2FC<0.35
p−value<0.05 & Log2FC>0.35
-Log10 P
Log2 fold change
C
group
control
Ang II
change
DOWN
UP
D
ATP−dependent activity, acting on DNA
regulation of synapse structure or activity
heme binding
cellular response to unfolded protein
heart growth
endoplasmic reticulum membrane
positive regulation of autophagy
regulation of mitotic cell cycle phase transition
regulation of metabolic process
signaling receptor activity
response to glucocorticoid
regulation of leukocyte mediated cytotoxicity
meiosis I
organic anion transmembrane transporter activity
monoatomic cation transport
cold−induced thermogenesis
regulation of mRNA splicing, via spliceosome
extracellular matrix organization
extracellular space
NES
Pathway size
E
logFC
KEGG Terms
Cellular senescence
p53 signaling pathway
IL−17 signaling pathway
Cell cycle
DNA replication
Phospholipase D signaling pathway
F
Enriched pathways
Phospholipase D signaling pathway
p53 signaling pathway
Neuroactive ligand−receptor interaction
IL−17 signaling pathway
Homologous recombination
Gap junction
Fanconi anemia pathway
DNA replication
Cytokine−cytokine receptor interaction
Choline metabolism in cancer
Chemokine signaling pathway
Cellular senescence
Cell cycle
Pathway
NES
−log10(adj_pval)

Figure EV2. The visualization of GSE47529 gene expression analysis results.

(**A**) The boxplots of normalized gene expression. (**B**) A volcano plot of differentially expressed genes, ($n = 7$). (**C**) DEG expression heat map of differentially expressed genes. (**D**) Enrichment network displaying the GO enrichment analysis results for GSE47529. (**E**) Visualization of selected KEGG pathways and their core enrichment genes in GSE47529. (**F**) Top 13 KEGG pathways enriched in GSE47529 DEGs.

© The Author(s)

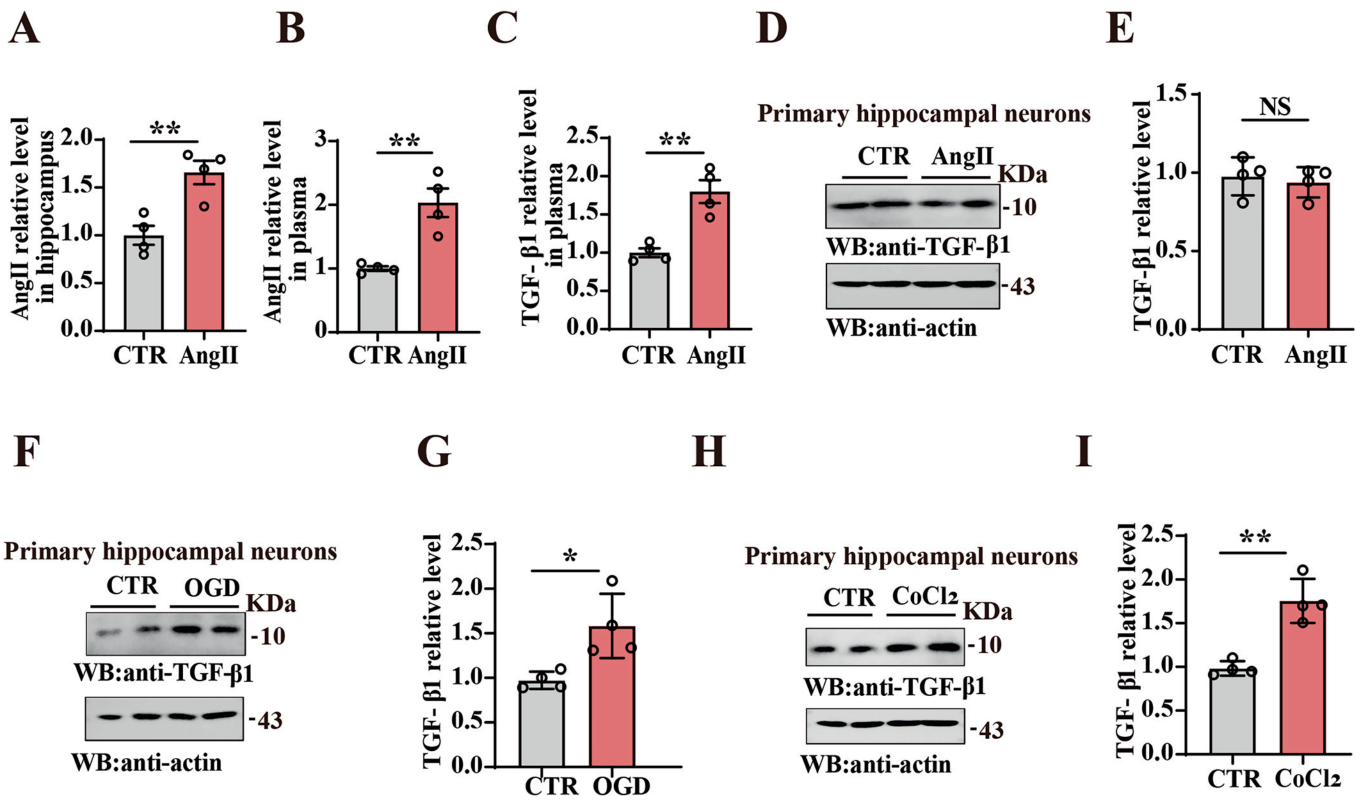

Figure EV3. The AngII and TGF-β1 levels were tested with different conditions.

The AngII levels were measured in hippocampus ($P = 0.0059$), ($n = 4$), (**A**) and in plasma ($P = 0.0038$), ($n = 4$), (**B**) in the two groups ($n = 4$). The TGF-β1 levels were measured in plasma ($P = 0.0025$), ($n = 4$), (**C**) in the two groups. (**D**) Hippocampal primary neurons were treated with compound AngII. Western blotting showed the TGF-β1 levels after treatment of hippocampal neurons with AngII 0.5 μM. Actin was used as a loading control. (**E**) Quantitative analysis of the TGF-β1 levels ($n = 4$). (**F**) Hippocampal primary neurons were treated with oxygen-glucose deprivation (OGD) for 12 h. Western blotting showed the TGF-β1 levels after OGD. Actin was used as a loading control. (**G**) Quantitative analysis of the TGF-β1 levels ($P = 0.0173$), ($n = 4$). (**H**) Hippocampal primary neurons were treated with $CoCl_2$ 100 μM for 6 h ($CoCl_2$ was a drug for chemical hypoxia model). Western blotting showed the TGF-β1 levels after $CoCl_2$. Actin was used as a loading control. (**I**) Quantitative analysis of the TGF-β1 levels ($P = 0.0011$), ($n = 4$). Data are presented as mean ± SD. A two-tailed Student's *T* test was used for statistical analysis in (**A–C**, **E**, **G**, **I**). $^*P < 0.05$, $^{**}P < 0.01$, versus Control group. Source data are available online for this figure.

© The Author(s)

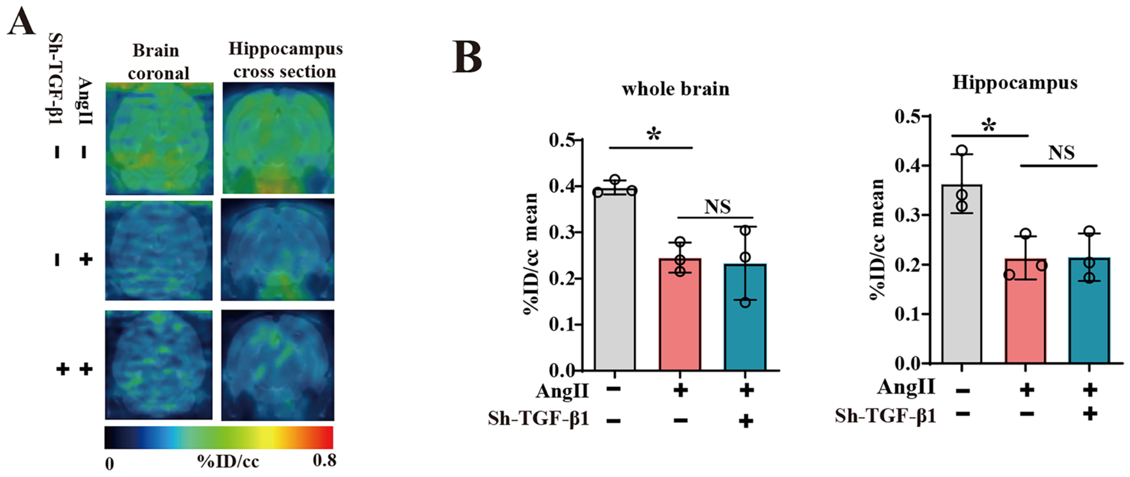

Figure EV4. Downregulation of TGF-β1 in AngII rats didn't change blood flow.

(**A**) The 13N-NH3-PET rat brain dynamic imaging was used to observe the blood flow in the brain and hippocampus: the whole brain ($P = 0.0122$) and the hippocampus ($P = 0.0256$) were measured, ($n = 3$). (**B**) Data are presented as mean ± SD. One-way ANOVA with Tukey's multiple comparisons test was used for statistical analysis in (**B**). *$P < 0.05$, versus AngII group. Source data are available online for this figure.

 © The Author(s)

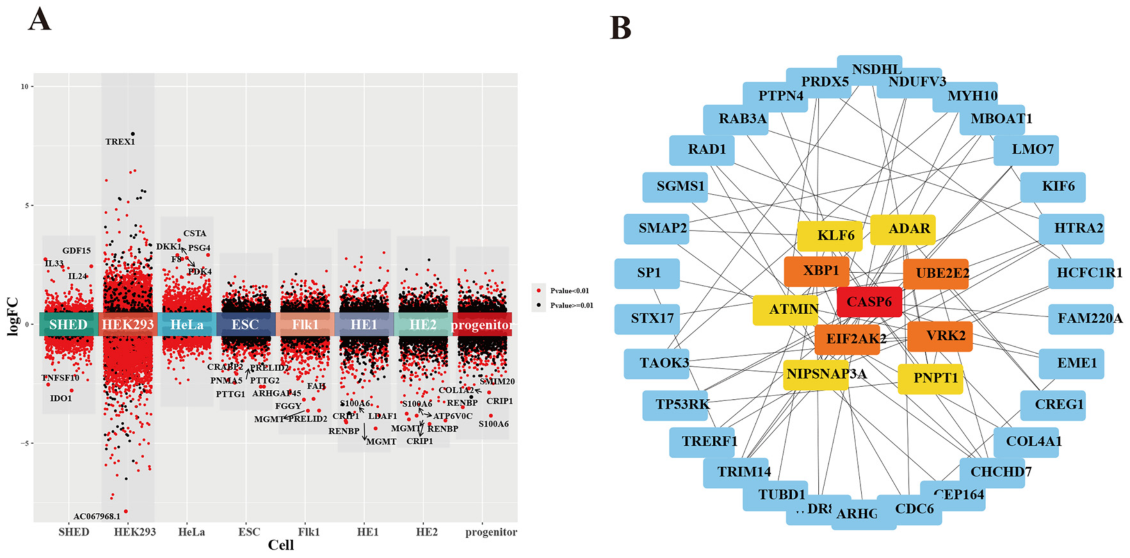

Figure EV5. The visualization of GSE126500, GSE165771, GSE37935, and GSE31628 gene expression analysis results.

(**A**) Scatter plot of Log2FoldChange (low Sp1 expression vs. high Sp1 expression) in different cell types, including SHED, HeLa, HEK293, ESC, Flk, HE1, HE2, and progenitor cells. (**B**) Protein–Protein Interaction (PPI) network and top 10 hub genes of the intersected genes.
